# Research Hotspots and Trends of Social Robot Interaction Design: A Bibliometric Analysis

**DOI:** 10.3390/s23239369

**Published:** 2023-11-23

**Authors:** Jianmin Wang, Yongkang Chen, Siguang Huo, Liya Mai, Fusheng Jia

**Affiliations:** 1 College of Arts and Media, Tongji University, Shanghai 201804, China; 2 Shenzhen Research Institute, Sun Yat-Sen University, Shenzhen 518057, China; 3 College of Design and Innovation, Tongji University, Shanghai 200092, China; 2210919@tongji.edu.cn (Y.C.);

**Keywords:** social robot, interaction design, bibliometric, VOSviewer, Citespace

## Abstract

(1) Background: Social robot interaction design is crucial for determining user acceptance and experience. However, few studies have systematically discussed the current focus and future research directions of social robot interaction design from a bibliometric perspective. Therefore, we conducted this study in order to identify the latest research progress and evolution trajectory of research hotspots in social robot interaction design over the last decade. (2) Methods: We conducted a comprehensive review based on 2416 papers related to social robot interaction design obtained from the Web of Science (WOS) database. Our review utilized bibliometric techniques and integrated VOSviewer and CiteSpace to construct a knowledge map. (3) Conclusions: The current research hotspots of social robot interaction design mainly focus on #1 the study of human–robot relationships in social robots, #2 research on the emotional design of social robots, #3 research on social robots for children’s psychotherapy, #4 research on companion robots for elderly rehabilitation, and #5 research on educational social robots. The reference co-citation analysis identifies the classic literature that forms the basis of the current research, which provides theoretical guidance and methods for the current research. Finally, we discuss several future research directions and challenges in this field.

## 1. Introduction

The social robot is a type of robotics technology that interacts with users in a natural and intuitive way by using the same social norms as humans, and typically is in an anthropomorphic form [1,2,3,4,5]. In recent years, these social robots have become increasingly prevalent in daily life, taking on tasks that were once performed exclusively by humans. These tasks include providing care for the elderly, accompanying children, offering entertainment resources, and facilitating online education [6,7,8]. This transformation of robots from service tools to social partners will profoundly impact economic and social dimensions [8,9]. Therefore, in order to fully integrate into the home and everyday environment, careful consideration of the future roles of robots and ensuring their perfect fulfillment of these roles through human–robot interaction design is of paramount importance [3,10,11]. Social robot interaction design (SRID) is the key to creating a positive user experience, establishing emotional connections, and adding value, which requires comprehensive research into the social behaviors, gestures, body movements, role design, and voice, among other aspects of robots, in a systematic manner [12,13,14,15]. In the long term, SRID serves as a crucial factor in ensuring the user acceptance and sustained use of social robots. Effective interaction design can transform robots into valuable partners for users, further fostering the development and innovation of human–robot relationships [16].

The literature review helps to further strengthen the framework of knowledge on a research topic by combing and evaluating relevant literature to identify research gaps [17,18]. Nevertheless, it is important to note that most of the current literature reviews on social robots tend to be either broad and unsystematic or narrowly focused on specific aspects of social robot research. Fewer studies have conducted a systematic literature review on social robot interaction design (SRID). For example, gesture design for social robots and its impact on human–robot interaction [19], robotic vision for human–robot interaction and collaboration [20], the design and evolution of social robots [21], the effect of social robot design features on the quality of human–robot interactions [22], cultural influences on user interaction with social robots [23], human–robot emotional interaction based on the perspective of robot psychology [24], empathy in human–robot interaction [25], facial anthropomorphic trustworthiness for social robot design [26], and children’s acceptance of social robots [27]. Furthermore, some studies focus on conducting reviews of specific types of social robots. These include educational social robots [7], elderly companion social robots [28], anxiety intervention social robots [29], child psychotherapy social robots [30], and robots for measuring children’s mental health [31]. Therefore, for social robot interaction design (SRID), there remains a notable absence of systematic literature reviews that could offer a comprehensive overview of the current research status, its theoretical foundations, and potential future research directions.

In the past decade, research in social robot interaction design (SRID) has experienced rapid growth, leading to a substantial body of literature on the subject [16,19,32]. Such a significant growth in the literature requires new approaches to review and analyze trends within knowledge domains [33,34]. In addition, SRID research exhibits a strong cross-disciplinary nature with a complex and diverse knowledge structure. Relying solely on traditional literature review methods makes it challenging to comprehensively grasp the current state of SRID research and the evolution of research hotspots [35,36]. Bibliometrics is an effective quantitative method for visualizing and analyzing the existing published literature. It enables the extraction of regular patterns and underlying insights from a large volume of papers, and is now widely employed in numerous scientific research fields [32,37]. Therefore, this paper will use bibliometrics to address the following five research questions to provide a comprehensive overview of SRID:

RQ1: What are the main focuses of SRID’s current research hotspots?

RQ2: How has the research content in SRID evolved over the past decade in terms of temporal development? What are the overall trends?

RQ3: What are the primary knowledge foundations and classical theories of SRID research?

RQ4: Which scholars and institutions worldwide are the research subjects of SRID? What are the collaborative relationships between them?

Clarifying these questions can help the relevant scholars grasp the research structure and latest development trends of social robot interaction design, and provide a corresponding knowledge base for further research in this field. Additionally, we explored research directions and applications that have received little attention in previous studies, which remain challenges for future research.

## 2. Research Design

### 2.1. Data Sources

Web of Science is the most comprehensive English language database in the world, and the literature in the Web of Science Core Collection has undergone peer review and rigorous scrutiny by publishing journals, so it is considered to possess greater disciplinary representativeness than other databases [38]. In order to ensure the validity and reliability of the research data, this study chooses the Web of Science Core Collection (WOSCC) as the data source. Our search terms combine “social robot” and “interaction design” to filter and obtain literature relevant to human–robot interaction design for social robots. Since there are many different expressions of relevant keywords in the English context, we list as many expressions as possible and connect these keywords with the Boolean operator symbol “OR”. The specific search strategy is TS = ((social robot OR social robotic OR social robots OR assistive robot OR conservational robot OR chatbot OR chat robot OR embodied conversational agent) AND (human robot interaction design OR interaction design OR collaborative design OR interactive design OR HRI design)).

To avoid the loss of interdisciplinary literature, the citation index sources were set to “All” (SSCI, SCI, A&HCI, CPCI-S, CPCI-SSH, BKCI-S, BKCI-SSH, and ESCI, among others) and the sources were not streamlined. Setting the time span as nearly 10 years (1 January 2014 to 25 July 2023), a total of 3379 initial documents were retrieved. As this study primarily focuses on empirical research, 179 review papers were excluded from the analysis. Subsequently, we reviewed the abstract of each paper to determine whether it met the criteria of this study. Among them, 761 papers were excluded due to their deviation from the research topic. Finally, 23 non-English papers were also excluded. As a result, 2416 relevant papers (from 2014 to 2023) were retained, and these were exported in TXT plain text format for bibliometric analysis. Among them, 1339 were journal articles, and 1077 were conference papers.

### 2.2. Research Methods

**Figure 1:**
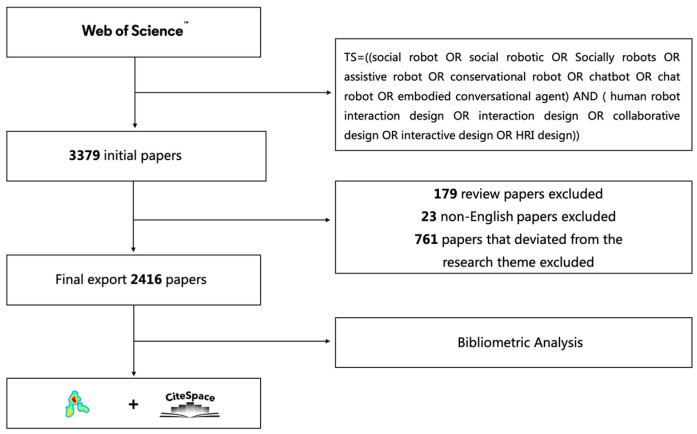
Research framework.

Bibliometrics is widely recognized as one of the primary methods for analyzing literature reviews, and has become a mainstream approach in scientific policy and research management in recent years [39,40]. Bibliometrics refers to the quantitative analysis of various types of literature to discover the patterns and potential information behind the literature data and then provide new insights into the current status and development trends of a specific research field. The concept was first introduced by Pritchard in 1969 [37]. Commonly used bibliometric software includes CiteSpace, VOSviewer, HistCite, and BibExcel. Each software has different functions and limitations [33,41]. Among them, CiteSpace and VOSviewer are both bibliometric analysis tools running on JAVA programs, and can effectively establish the mapping relationship between the knowledge units of the literature and visualize the macrostructure of the knowledge through knowledge mapping [41]. CiteSpace provides various vital indicators, such as keyword burst and time zone, which can visually identify the development trend of a specific research area and the evolution process of knowledge [34]. VOSviewer provides text mining function to build knowledge maps based on metrics such as co-occurrence, bibliographic coupling, and co-cited references [33]. In order to obtain more rigorous and comprehensive analytical indicators, Our study used two bibliometric visualization tools, VOSviewer (V1.6.19) and CiteSpace (V6.2.R4), to conduct a comprehensive analysis of SRID research, to explore the knowledge structure and development trend of the SRID research, and further discuss possible future research priorities. Figure 1 illustrates the research framework of this study.

## 3. Bibliometric Results and Analysis

### 3.1. Trend Analysis of Annual Outputs of SRID Literature

**Figure 2:**
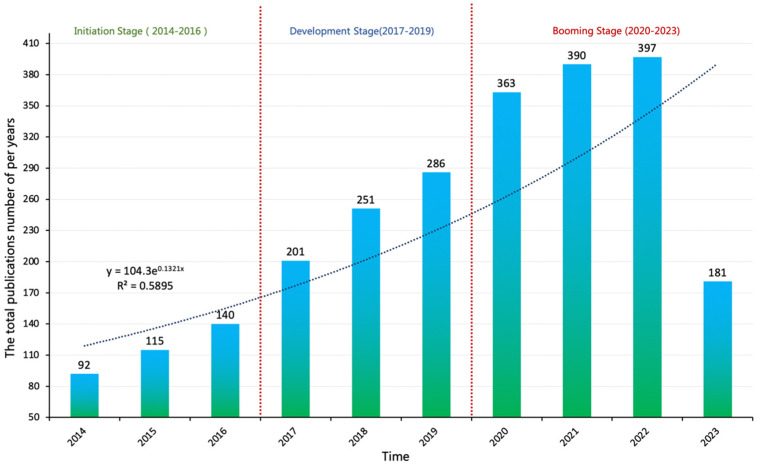
A distribution map of the annual publication volume of SRID.

Statistical patterns of change in the output of academic literature over time can effectively assess the accumulation of knowledge, research dynamics, and maturity level of a discipline [18,42]. The annual distribution curve of the literature output in the SRID is shown in Figure 2. The distribution of literature outputs in Figure 2 demonstrates a consistent upward trend in the number of publications, which shows that the research topic of SRID is constantly evolving and has been the focus of researchers’ attention in recent years. Based on the trend of literature output in the last decade, the entire development of SRID research can be categorized into three main stages: the initial stage (2013–2016), the developmental stage (2017–2019), and the booming stage (2020–2023). As shown in Figure 2, the literature production of SRID research was relatively low during the initial stage (2013–2016), with an average annual publication volume of 115 papers per year, and the annual publication count did not exceed 150 papers. Starting in 2017, this field entered a stage of rapid development, with an average annual publication volume of 246 papers published per year and a rapid growth rate. From 2020 to 2023, with the development and popularization of technologies such as artificial intelligence, affective computing, and cloud computing, the literature output in this field began to sharply increase. By 2023, the average annual number of publications was as high as 332 per year, the growth rate was more stable, and SRID research entered a prosperous stage.

### 3.2. Research Hotspots of SRID

**Figure 3:**
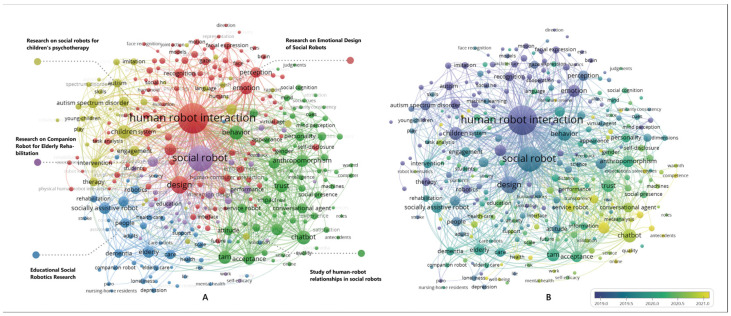
(**A**,**B**) keyword co-occurrence clustering.

A paper’s keywords serve as a high-level summary of the authors’ research results, typically encompassing research objects, perspectives, and methods. High-frequency co-occurring keywords reflect the long-standing research hotspots of SRID [37,43]. The 2416 documents within the search contained a total of 6889 keywords. In order to ensure the consistency of keyword expressions and the clarity of homonyms, keywords were standardized and synonyms were merged based on suggestions from Frey and Dueck, following discussions among three senior professors and the research team [44]. For example, “Customer Experience” was replaced with “User Experience”, “ASD” and “Autism Spectrum Disorders” were replaced with “Autism Spectrum Disorder”, and “Older Adults”, “Elderly-People”, “Aging”, etc., were replaced with “Elderly”. Importing the WOS data into VOSviewer, we set a co-occurrence frequency threshold of more than 8 to ensure the quality of the plots. After merging synonymous keywords, the resulting keyword co-occurrence clustering diagram for SRID research is shown in Figure 3A. There are 288 keyword nodes, 9560 connecting lines in the figure, and keywords with the same color in the graph are the same clusters, which form a total of 5 major clusters. That is, the current research of SRID mainly focuses on #1, the study of human–robot relationships in social robots, #2, research on the emotional design of social robots, #3, research on social robots for children’s psychotherapy, #4 research on companion robots for elderly rehabilitation, and #5 research on educational social robots. Figure 3B is a keyword co-occurrence cluster–time overlay diagram reflecting the temporal distribution of these five SRID research clusters. Table 1 provides detailed information about the SRID research clusters, and the following sections outline the specific research content within these clusters.

**Cluster #1**—the study of human–robot relationships in social robots, which consists of 101 keywords, mainly including acceptance, anthropomorphism, attitude, trust, privacy, self-disclosure, satisfaction, TAM (Technology Acceptance Model), social presence, warmth, transparency, roles, experience, and other keywords. The human–robot relationship is an essential topic in SRID research and is an emerging research theme (see Figure 3B). Furthermore, as robots become highly intelligent, people will naturally and unconsciously apply the social rules of interpersonal communication to human–robot interactions, even if the robots are not humans [45]. Scholars such as Nass use the “Computer is a Social Actor (CASA paradigm)” to describe this phenomenon, and social robots’ behaviors are particularly easy to perceive as social behaviors due to their anthropomorphic design and autonomous behaviors [46,47]. The research content of the human–robot relationship mainly includes human–robot trust (trust), robot acceptance (acceptance), user self-disclosure willingness (self-disclosure), user satisfaction (satisfaction), and user privacy concerns (privacy) [10,48]. This direction of research is mainly based on the user’s psychological cognition, through the use of the model research method (structural equation model) and user experiments to conduct quantitative empirical research, in order to identify the influencing factors affecting human–robot relationships, as well as the influence mechanism [14,49,50]. From the large number of research results accumulated in the current literature, the influencing factors (antecedent variables) of the human–robot relationship can be broadly categorized into three dimensions: “Human”, “Robot”, and “Environment” [51,52,53]. For example, the keywords “Anthropomorphism”, “Roles”, “Appearance”, “Transparency”, “Social Presence”, and “Warmth” appearing in Cluster #1 are antecedent variables belonging to the “Robot” dimension [52,54,55,56,57]. Combined with the characteristics of high-frequency keywords, the research focus of Cluster #1 can be further summarized as building a more harmonious social human–machine relationship through the interaction design of social robots.

**Cluster #2**—Research on the emotional design of social robots. This cluster comprises 60 cluster members, primarily including keywords such as affective computing, affective robotics, emotion, emotion recognition, facial expression, face, body language, EEG, brain, eye contact, gaze, gesture recognition, and more. Emotional design for social robots refers to imbuing robots with emotions, enabling them to recognize better, understand, and respond to users’ emotions through emotional expressions [2,19,48]. A social robot with effective emotional design can enhance the interactive experience of human–robot interaction, improve user satisfaction, and facilitate a more natural and enjoyable communication process [2,58]. Specifically, emotional recognition in social robots refers enabling robots to recognize and understand human emotional expressions, allowing the robots to perceive and appropriately respond to users’ emotions during interactions [59]. The emotional expression design for social robots primarily includes two categories: verbal cues and non-verbal cues. Verbal cues encompass the tone, intonation, and conversational style of social robots’ speech, while nonverbal cues encompass facial expressions, gestures, body movements, and gaze, among others [2,50]. For example, robots can use gestures, natural language, and even “eye contact” to engage in positive emotional interactions with users [19,49,60]. As shown in Figure 3B, the average occurrence time of keywords in this cluster is relatively early, which belongs to the research basis of human–computer interaction design of social robots. From the perspective of comprehensive keyword characteristics, this cluster primarily delves into the impact of social cues and emotional factors on human–robot interaction, focusing on the recognition and expression of social robot emotions and their integration into specific designs and evaluations [2,49,58]. 

**Cluster #3**—Research on social robots for children’s psychotherapy is composed of 51 keywords such as autism, therapy intervention, children, anxiety, training, social skills, spectrum disorder, games, and speech. This cluster focuses on the specific impact of the interaction design of social robots on the effectiveness of children’s psychotherapy and emotional experience [61]. The psychotherapy social robot provides targeted interventions by analyzing children’s psychological needs and emotional states, and is specifically designed to alleviate negative emotions such as anxiety and depression [62]. A large number of studies have demonstrated that social robots have advantages in children’s psychotherapy compared with other therapeutic methods [30], such as avoiding the psychological barriers that may arise from personal emotions and social stigma in traditional psychotherapy scenarios, not being affected by time and location restrictions, not being limited by the number of psychologists, and higher acceptance and enjoyment in interactions [63,64,65,66]. From the high-frequency keywords in the cluster, social robots are also often used in psychotherapy scenarios, such as in therapy for children with autism spectrum disorders and social skills training. Typical psychotherapy social robots include Woebot, Wysa, Joyable, and so on. These robots are used in more than 130 countries around the world, and have been shown to be effective through psychological evaluations [67,68]. Nonetheless, this research direction is still in its early stages. Future investigations must address fundamental research questions related to children’s perception, cognition, behavior, and psychotherapy, among other aspects [21,30,69]. Furthermore, many current studies lack rigorous quantitative statistical analysis, and there is a relatively limited number of child participants in user experiments [70].

**Cluster #4**—Research on companion robots for elderly rehabilitation mainly contains 48 keywords such as elderly, health-care, loneliness, mental health, assisted therapy, co-design, depression, user-centered design, and others, and at the same time presents a very inclusive perspective. This cluster adopts an inclusive perspective and primarily focuses on the research of interaction design for rehabilitation companion robots. These robots aim to support the independent living of elderly individuals in their homes, meeting their real-world needs, promoting physical and mental health, and preserving their dignity, privacy, and independence [55,71]. Numerous studies have shown that assisting elderly individuals with physical and cognitive training through social robots has a significant positive impact on their cognitive function, overall well-being, and quality of life [72,73]. For instance, it leads to a reduction in diseases such as diabetes, Alzheimer’s disease, and cardiovascular conditions among the elderly population. In this research scenario, the social robot mainly acts as an exercise partner or trainer to provide social interaction and companionship for the elderly [74]. To maximize user enthusiasm and engagement, robots should be user-centered, adapting their interaction strategies based on the personalized needs, actual abilities, and daily habits of the elderly [28,74,75].

**Cluster #5**—Educational social robotics research contains a total of 28 cluster members, mainly containing the keywords “Educational Robot”, “Performance”, “Knowledge”, “Teacher”, “Classroom”, “Child-Robot Interaction”, “Cognition”, “Empathy”, “Interaction Design”, and other keywords. Educational social robots are designed to provide educational and learning support by interacting naturally with students, developing logical thinking skills, language expression abilities, teamwork ability, creativity, and more [7,76]. In learning environments, these robots typically play roles such as tutoring teachers, learning partners, and information providers [76,77]. The research topics mainly include user interface design, dialogue language design, emotional interaction design, and personalized learning support, among other aspects, and involve interdisciplinary knowledge such as education, psychology, and computer science [7,61,78]. Combining the characteristics of high-frequency keywords, the research hotspots in Cluster #5 can be succinctly summarized as the investigation of design strategies for the interaction between social robots and student users. The aim is to provide effective learning support and create a more enjoyable user experience.

### 3.3. The Evolution and Trend of SRID Research Hotspots

**Figure 4:**
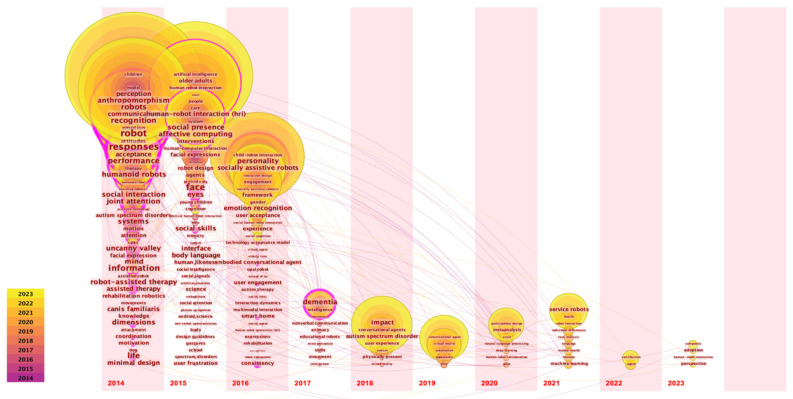
Keyword time zone view of SRID.

**Figure 5:**
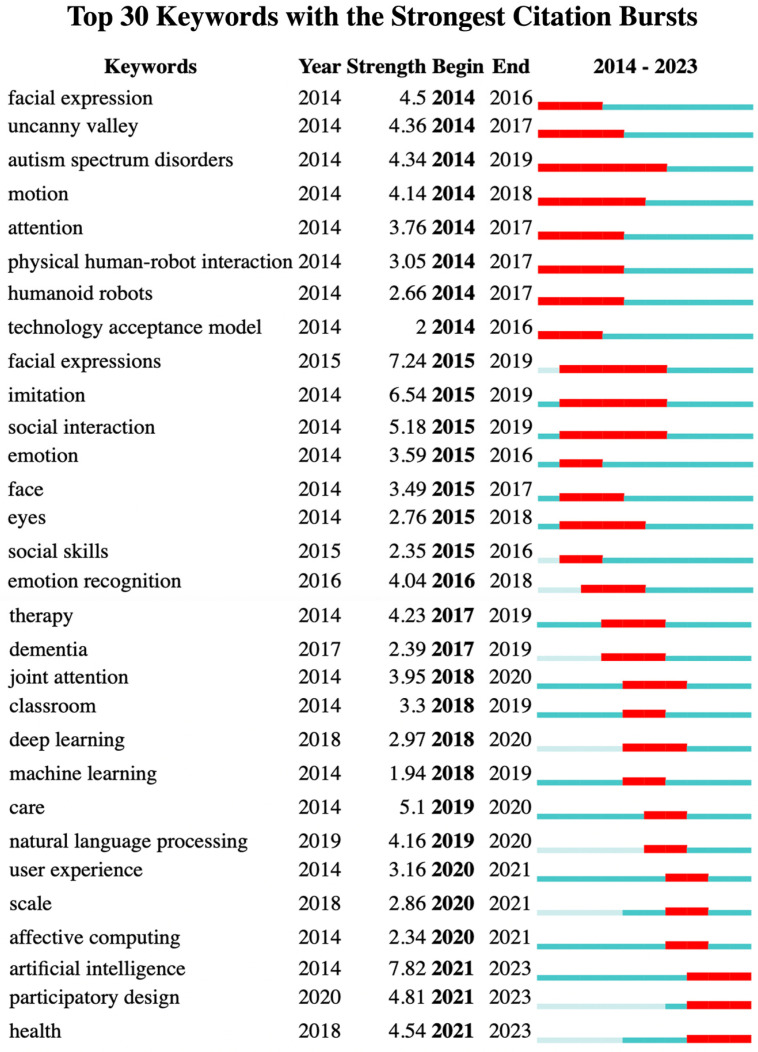
Keyword burst.

To better understand the evolution and development trends of SRID research hotspots, we conducted a statistical analysis of the average appearance time of keywords, resulting in Figure 4 (keyword: time zone) and Figure 5 (burst keywords’ emergence). The time zone map intuitively reflects the frequency size and first appearance time of keywords within the search range, and is often used by scholars to identify the previous trend of research topics. Figure 5 lists the top 30 keywords and their emergence intensity in different periods, where the darker parts characterize the intensity of keyword bursts in the literature and the years in which they were relatively prominent. The burst keywords represent the level of attention of research topics in different periods and can also reflect the changing trends and past research hotspots in the field [79]. The time zone and burst keywords combine keywords with the time dimension for analysis. By combining the insights from the time zone and burst keywords, we aim to obtain more robust research results. 

In Figure 4, it is evident that early SRID research focused on appearance design for social robots, with keywords like anthropomorphism, uncanny valley, humanoid robots, and facial expressions. Subsequently, the research started to turn to social cues of emotional design, such as gestures, body language, affective computing, face, eyes, etc. The subsequent research focused more on specific application scenarios, such as psychotherapy, rehabilitation assistance, and companion learning. The keywords after 2020 are human–robot collaboration, machine learning, deep learning, natural language processing, satisfaction, adoption, individual differences, participatory design, and more. It can be seen that the research focus is gradually shifting to human–computer collaboration and human–computer relationship research, and, at the same time, the research methods are also more cutting-edge, and the integration of traditional research methods with new technologies such as artificial intelligence is more frequent. 

The top 30 burst keywords in Figure 5 provide further insights, revealing the transition from early keywords like facial expressions and uncanny valley to emotion recognition, and onwards to affective computing, artificial intelligence, and participatory design. This trend underscores the shift from embodied interaction design research to human–robot relationship research. Combining the insights from the time zone analysis, burst keywords, and the co-occurrence cluster map, it is evident that the future of SRID research will be centered around several key areas. These include human–robot collaboration, affective computing, artificial intelligence, human–robot trust, user self-disclosure, emotion recognition and expression, and interaction design evaluation. This synthesis of research trends underscores the anticipated focus on advancing the understanding of how social robots can effectively collaborate with humans, integrate emotions, leverage AI capabilities, foster trust, facilitate user self-disclosure, and enhance interaction design evaluation. This comprehensive perspective offers valuable guidance for researchers, practitioners, and policymakers shaping the trajectory of SRID research and its future applications.

### 3.4. Knowledge Base of SR-HRI Research

**Figure 6:**
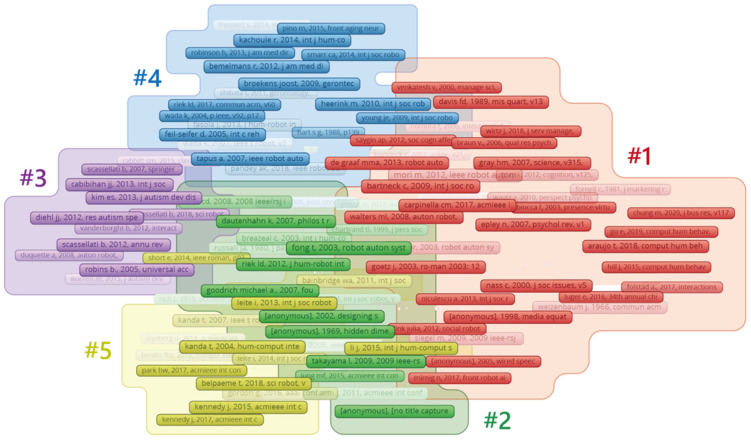
Reference co-citation cluster network.

References form a co-citation relationship due to being cited in pairs in the citing documents, and the higher the co-citation strength between two papers, the more significant the correlation between them [80]. The co-citation frequency of a reference indicates its influence and importance in the research field [81,82]. This indicator can be used to understand which papers in a research field have received extensive attention and citations and which works have had a significant impact on the development of the field [32]. To better comprehend the knowledge structure and research foundation of SRID, we performed a reference co-citation analysis. According to the statistics, 75,022 valid references from 44,524 scholars were cited in the 2416 papers within the search scope. Based on VOSviewer, we extracted references with a citation frequency of no less than 20 in 2014–2023. We generated a reference co-citation cluster network of 300 references and 19,944 co-citation relationships, as shown in Figure 6. Consistent with the results of the keyword co-occurrence cluster, the reference co-citation cluster still forms five clusters: #1 the study of human–robot relationships in social robots, #2 research on the emotional design of social robots, #3 research on social robots for Children’s psychotherapy, #4 research on companion robots for elderly rehabilitation, and #5 research on educational social robots. The papers in these five clusters constitute the most critical knowledge foundation in the field of SRID, connecting the research contents of different disciplines.

In Cluster #1 (the study of human–robot relationships in social robots), the article “Measurement Instruments for The Anthropomorphism, Animacy, Likeability, Perceived Intelligence, and Perceived Safety of Robots”, published by C Bartneck et al. in 2009 in the *International Journal of Social Robotics*, received the most attention, with a total of 201 citations in our co-citation network. In contrast to traditional reviews, this study conducted a systematic literature review on anthropomorphism, animacy, likeability, perceived intelligence, and perceived safety, which are the five key concepts of social robot design. It also examined measurement methodologies and refined the results into five semantic difference scales with high internal consistency. This work serves as the conceptual basis for studying human–robot relationships in social robots [83]. The second most cited paper is “Anthropomorphism and The Social Robot” by BR Duffy, published in *Robotics and Autonomous Systems* in 2003. The paper delves into the mechanisms underlying anthropomorphism and introduces the critical social features necessary for robots to engage in social interactions. It also discusses how anthropomorphic design can make it easier for users to comprehend and justify their actions [10]. In addition, the paper “Machines and Mindlessness: Social Responses to Computers”, published by Clifford Nass et al. in 2000, has also attracted much attention. This paper further validates the CASA paradigm by reviewing a series of experimental studies, proves that the depth of social response is related to the “personality” of the computer, and explains the psychological cognitive process behind this phenomenon. This paper represents the second generation of research within the CASA paradigm, which focuses on explaining the social responses generated in human–robot interactions to advance further research on human–robot relationships [47].

Cluster #2 (research on the emotional design of social robots) consists mainly of classic literature related to emotional design of social robots. At the center of the cluster is “Emotion and Sociable Humanoid Robots”, a 2003 paper by C Breazeal, which focuses on the critical role of emotional expressive behavior in social interactions between users and anthropomorphic social robots. The paper takes the Kismet robot as an example to carry out the development and verification work, puts forward the key principles in the theory of emotion and its expression, and establishes the scientific basis for the emotion model and expression behavior of social robots. LD Riek’s 2012 article (the second most cited at 65 citations) systematically reviewed 54 HRI Wizard of Oz experiments and proposed new experimental reporting standards to guide researchers to adopt more rigorous methodology to conduct and report HRI experiments and obtain more convincing research conclusions [84]. The article “A Circumplex Model of Affect”, published by Professor James Russell in 1980, was the third most frequently cited article. This article proposed the emotional space model (CMA) to visualize the mechanism and process of emotional generation. In this model, emotional concepts such as pleasure (0°), arousal (45°), excitement (90°), distress (135°), displeasure (180°), depression (225°), sleepiness (270°), and relaxation (315°) are arranged in a circular order. The model provides follow-up researchers with an expressive way to represent and assess the interrelatedness of emotional dimensions [85].

In Cluster #3 (research on social robots for children’s psychotherapy), the article “Keepon: A Playful Robot for Research, Therapy, And Entertainment” is the most influential, and is in the center of the co-citation network. The article designs and develops a Keepon child psychotherapy robot and longitudinal field observations of typical preschool children and children with autism spectrum disorder using the modified robot. The results of the qualitative and quantitative analyses suggest that the Keepon robot can help children understand socially meaningful information and motivate them to share it. These findings also emphasize the significance of interaction style in establishing human–robot relationships [86]. Second is the 2005 paper by B Robins et al. in *Universal Access in The Information Society*, which describes a long-term longitudinal study of four children with autism spectrum disorder. Quantitative analyses showed an increase in the duration of predefined behaviors in the later experiments, while qualitative analyses indicated a further increase in social interaction skills (imitation, turn-taking, and role-switching) and communicative competence demonstrated by children in an interactive setting. The findings also confirm the necessity and benefits of long-term longitudinal studies of HRI [87]. Subsequently, a paper by ES Kim et al. was published in the *Journal of Autism and Developmental Disorders* in 2013. This paper examined the effects of interacting with three different objects, (1) an adult, (2) a touch-screen computer game, and (3) a social robot, on interaction outcomes in 4–12-year-old children with autism spectrum disorders (ASD; N = 24). The results indicated that children said more words when the interaction partner was a robot compared to a human or computer game partner, and the study makes a strong case for the great potential of social robots in developing social skills and psychotherapy [88].

In Cluster #4 (research on companion robot for elderly rehabilitation), the most highly cited paper is “Assessing Acceptance of Assistive Social Agent Technology by Older Adults: The Almere Model.” The article extended the Technology Acceptance Model and the UTAUT model to test the intention of older users to use assistive robots, with the model incorporating function-related variables (such as perceived usefulness and perceived ease of use) and social interaction-related variables to explain the use intention. Three different elderly robots were tested using controlled experiments and longitudinal data, and the results showed strong support for the model, accounting for 59–79% of the variance in intention to use and 49–59% of the variance in actual use [89]. In a 2007 paper by K. Wada et al., two therapeutic robots were introduced into an elderly care facility for a long-term follow-up experiment to investigate the psychological and social impact of robots. The results showed that interaction with the robots increased their social interactions. In addition, physiological measurements showed that the subjects’ vital organ responses to stress improved after the introduction of the robots [90]. The 2013 paper by J Fasola et al. discusses the design methodology and implementation details of a Social Assistive Robot (SAR) system to help older adults participate in physical activity, includes insights from psychological research into intrinsic motivation, and proposes five clear SAR-based therapy design principles for interventions. A multi-session user study of the elderly (N = 33) also demonstrated the effectiveness of the SAR exercise system in terms of factors such as fun and social attractiveness [91].

In Cluster #5 (research on educational social robots), the review paper “Social Robots for Education: A Review” by T Belpaeme et al. was the most influential. This paper presents three critical studies by answering the following questions: (1) What is the effect of robot tutors in achieving learning outcomes? (2) How do the appearance and behavior of robots affect learning outcomes? (3) What are the potential roles of robots in educational settings? The paper provides a comprehensive overview of the application of educational social robots [92]. The second most highly cited paper in Cluster #5 is titled “Interactive Robots as Social Partners and Peer Tutors For Children: A Field Trial.” The paper reports an 18-day field experiment in a Japanese elementary school to investigate how educational robots establish relationships with children. The experimental results suggest that robots may be more successful in establishing common ground and influence when children already have some initial proficiency or interest in English. These results suggest that the design of interactive robots should have something in common with their users, providing both social and technical challenges [93]. In addition, C Bartneck’s 2004 paper “A Design-Centered Framework For Social Human-Robot Interaction” has also attracted much attention. The paper proposes a framework for categorizing attributes of social robots such as form, morphology, social norms, autonomy, and interactivity, and provides extensive guidelines for designing social robots [94].

### 3.5. Distribution of SRID Literature Sources

Analyzing the citations and the number of papers of the literature sources can help researchers understand which journals/conferences are the literature sources that contribute the most to the SRID research field [95]. Citations refer to the sum of the total citations of articles published in the journal within the scope of search, while the average number of citations is the average number of citations of articles published in the journal. According to the statistics, the 2416 valid documents within the scope of retrieval were published in 909 journals and conferences. Table 2 lists the top 10 high-impact journals/conferences in terms of total citations in the last decade, which account for 17.136% of the total number of publications. Among them, the *International Journal of Social Robotics* is the journal with the most papers in this research field, containing 184 papers, and it also has the highest total citations, reflecting its significant influence in the field of human–robot interaction design for social robots. Next is *Computers in Human Behavior*, with 39 publications and a total of 1268 citations. Ranked third is *Frontiers in Psychology*, with 34 publications and 764 total citations. These journals/conferences come from different research fields, such as robotics, psychology, computer science, management, geriatrics, and more. It can be seen that SRID is a key research topic with solid interdisciplinary characteristics, involving knowledge from multiple subject areas. According to the disciplinary statistical analysis of the WOS system, the 2416 documents are related to 83 major disciplines, and the top ten disciplines in terms of the number of publications are computer science, robotics, engineering, automated control systems, psychology, business, economics, education, neuroscience, sociology, and telecommunications. These disciplinary subjects are important research areas for SRID, providing both theoretical foundations and methodological tools for SRID studies. Furthermore, SRID also provides valuable development opportunities for advancements in these disciplines. In addition, journals such as the *International Journal of Human-Computer Studies*, *ACM Transactions on Human-Robot Interaction,* and *Interaction Studies* also have a significant influence on SRID research.

### 3.6. High-Impact Countries and Research Institutions

**Figure 7:**
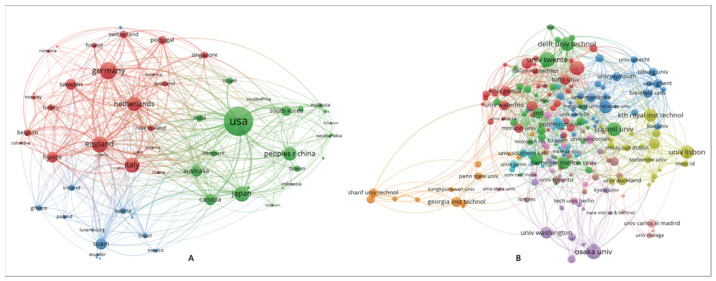
(**A**) is national cooperation network, (**B**) is institutional cooperation network.

As seen in Figure 7A, a total of 80 countries/regions around the world have contributed to the research field of SRID, and three cooperative communities have been formed in terms of cooperation, dominated by the United States, Germany, and Spain. Among these, the United States is the most influential country, with the highest number of publications and total citations (642 publications, 9398 total citations), Followed by Germany (247 articles and 2747 citations), the United Kingdom (216 articles and 3719 citations), China (198 articles and 1647 citations), Japan (196 articles and 1791 citations), Italy (186 articles and 1884 citations), New Zealand (179 articles and 2535 citations), Australia (114 articles and 1288 citations), Canada (112 articles and1491 citations), and Spain (109 articles and 958 citations). The publications of the top 10 countries accounted for 63.286% of the total number of publications, and their total citation counts are all above 100, which is an essential source of output for SRID research worldwide.

A total of 1959 research organizations worldwide have conducted SRID-related research in the last decade. Running VOSviewer and selecting organizations to set the node threshold to 5 yields a collaborative network of institutions with an annual node count of 218, as shown in Figure 7B. In terms of total citations, the top five research institutions were Stanford University (876 citations) followed by the Massachusetts Institute of Technology (MIT) (781 citations), University of Auckland (761 citations), Hertfordshire University (707 citations), and the University of Twente (682 citations). In terms of publications, the top five research institutions are the University of Twente (41 papers) followed by Osaka University (39 papers), Eindhoven University of Technology (38 papers), Cornell University (36 papers), and Delft University of Technology (35 papers).

### 3.7. High-Impact Author Analysis

Through author co-citations, we can study the more active scholars in the field of SRID research around the world [96,97]. A total of 7053 authors within the search scope have contributed to the research field of SRID. Table 3 lists the top five authors with high literature production and their respective total citations. Among them, Professor Paiva, Ana from the Polytechnic University of Lisbon published the most papers, with an H index of 43 and a total of 476 citations, and is ranked first with 30 publications within the search scope. Professor Paiva, Ana is followed by Hiroshi Ishiguro (Osaka University) with 29 papers and a total of 369 citations, Kerstin Dautenhahn (University of Waterloo) with 26 papers and 599 citations, Selma Sabanovic (Indiana University) with 24 papers and 237 citations, and Guy Hoffman (Cornell University) with 21 papers and 483 citations. In addition, among the high-yield authors, Professor Broadbent, Elizabeth of the University of Auckland had the highest number of total citations, with 13 papers published within the search scope and a total of 729 citations, followed by Breazeal, Cynthia (16 papers and 654 total citations), Dautenhahn, Kerstin (26 papers and 599 total citations), Mutlu, Bilge (18 papers and 550 total citations), and Guy Hoffman (21 papers and 483 total citations). These high-impact authors are considered pioneers and significant contributors to SRH-RID research.

## 4. Discussion

In the previous section, we identified the hot topics in the field of SRID research, the classic foundational literature, and the research collaboration network, etc. In this section, we discuss the existing challenges and future research directions.

(1) More Realistic Research Scenarios: Current research is still dominated by laboratory studies, lacking research in real and complex natural interaction scenarios, and the results obtained in laboratory environments may have limitations in terms of real-world applications. Research conducted in close-to-real-world contexts can help obtain more meaningful results and provide deeper insights into human behavior and robot interactions [98,99].

(2) Effective Measurement of Research Indicators: Current research presents difficulties and challenges in obtaining reliable data to evaluate human–social robot interactions. For example, the commonly used questionnaires are not well suited to young children, and the measurement of some variables is easily affected by the state of the subject and the external environment, such as trust and user emotional state [19,30]. Therefore, there are still some challenges in how to effectively and objectively measure the true level of such latent variables and improve the reliability, validity, and replicability of the data. This may require larger sample sizes, a more diverse set of approaches for measuring variables, and richer data types [22,46,100].

(3) Longitudinal Trial: Current research is mainly based on short-term experiments, while interactions between humans and social robots often occur over the long term. Short-term experiments cannot capture the long-term effects and how human–robot interaction changes over time [101]. For example, in the case of social robots used for psychotherapy and online education, the evaluation of learning and psychotherapy effects is a long-term process. Future research could conduct more long-term longitudinal trials to provide more comprehensive data to support the conclusions, thus providing insights into the phenomenon and its mechanisms changing over time [102,103].

(4) More Specific Design Strategies: Although the interaction design of social robots has accumulated a significant number of research findings, the current research is relatively scattered and has not yet formed a comprehensive design framework [70,104,105]. Taking the research on psychotherapy social robots as an example, the current research focuses on the acceptance, effectiveness, and feasibility of robot intervention, while the basic research related to the robot interaction design and the user’s emotional experience is relatively lacking, and the mapping mechanism between the interaction design factors and the healing effect is unclear. Therefore, in the future, it is necessary to study further the specific design features and specific design strategies of social robot interaction design, especially how the combination effect of different interaction design features will affect the efficiency of human–computer interaction and emotional experience [106].

(5) Uncovering the Psychological Cognitive Mechanisms behind User Behavior: There is a close interplay between a user’s psychological cognitive process, their experience, and their behavior [107,108,109]. Understanding the psychological cognitive process of users can help design social robots that better meet user needs and elicit positive emotions [110,111]. The current empirical research usually only gives general conclusions, and lacks empirical studies that explain how the interaction design of social robots affects user perception and user behavioral responses from the perspective of user psychology and cognition [112,113,114]. Therefore, this will also be a future research direction.

## 5. Conclusions

This paper provides a comprehensive review of the literature related to social robot interaction design research in the last decade based on bibliometrics, with the main contributions of (1) identifying the critical research hotspots and emerging trends in this field over the past decade; (2) identifying the classic literature, as well as the leading countries, journals, and authors that make up the knowledge structure of the field; and (3) discussing the challenges that the current social robot interaction design research is facing in terms of more authentic research scenarios, effective metrics for research indicators, longitudinal experimental design, more specific design strategies, and mining the mechanisms behind interaction behaviors, and presents potential research directions and opportunities. These findings can help scholars in this field gain a better understanding of the research structure and latest trends in social robot interaction design, and provide a solid foundation for further development.

## 6. Limitations and Future Research

To the best of our knowledge, this is the first bibliometric analysis of social robot interaction design. Unlike narrative reviews, scientific bibliometrics offer a comprehensive view of the field’s historical development and emerging trends, aiding HRI scholars. Despite this study’s great potential and the fact that it has provided valuable new insights, certain limitations must be considered. Firstly, in terms of data sources, although we chose the most representative Web of Science core collection as the data source for our study, relying on a single database may bias our findings. Therefore, in the future, it is necessary to use data from more databases for supplementary research, such as using related publications from other databases such as “Scopus”, to make the conclusions more comprehensive [41]. Secondly, our analysis focused solely on journal and conference papers in English, excluding “books” and “dissertations”. Subsequent research could explore SRID using a broader range of data sources. Lastly, the co-citation networks may not fully capture the most recent trends, as recent publications may not have accumulated sufficient citations.

## Figures and Tables

**Table 1:** Specific information on SRID’s 5 research clusters.

Cluster	Cluster Label	Average Time	Keywords Number	Keywords
1 (green)	Study of human–robot relationships in social robots	2021	101	Acceptance; anthropomorphism; attitude; trust; privacy; self-disclosure; TAM; warmth; transparency;
2 (red)	Research on emotional design of social robots	2019	60	Affective computing; affective robotics; emotion; facial expression; body language; eeg; gaze; gesture recognition;
3 (yellow)	Research on social robots for children’s psychotherapy	2019	51	Autism; therapy intervention; children; anxiety; training; social skills; spectrum disorder; games; speech
4 (blue)	Research on companion robots for elderly rehabilitation	2020	48	Elderly; health-care; loneliness; mental health; assisted therapy; co-design; depression; user-centered design
5 (purple)	Educational social robotics research	2020	28	Educational robot; performance; knowledge; teacher; classroom; child-robot interaction; empathy; interaction design

**Table 2:** Distribution of SR-HRI literature sources (top 10).

Ranking	Literature Source	Publisher	Citations	Number of Publications	Average Number of Citations
1	*International Journal of Social Robotics*	SPRINGER	4010	184	21.793
2	*Computers in Human Behavior*	ELSEVIER	1268	39	32.512
3	*Frontiers in Psychology*	FRONTIERS	764	34	22.470
4	*International Journal of Human-Computer Studies*	ELSEVIER	734	22	33.363
5	ACM/IEEE International Conference on Human-Robot Interaction (HRI2015)	ACM	563	12	46.916
6	*International Journal of Contemporary Hospitality Management*	EMERALD	544	7	77.714
7	*Journal of The American Medical Directors Association*	ELSEVIER	440	4	110.034
8	ACM/IEEE International Conference on Human-Robot Interaction (HRI2018)	ACM	435	13	33.461
9	*Frontiers in Robotics and AI*	FRONTIERS	388	69	5.623
10	ACM/IEEE International Conference on Human-Robot Interaction (HRI2019)	ACM	363	30	12.021

**Table 3:** Top 5 high-yield authors.

Ranking	Author	Institution	Number of Published Papers	Number of Citations	H Index
1	Paiva, Ana	Polytechnic University of Lisbon	30	476	43
2	Ishiguro, Hiroshi	Osaka University	29	369	54
3	Dautenhahn, Kerstin	University of Waterloo	26	599	56
4	Sabanovic, Selma	Indiana University	24	237	32
5	Guy Hoffman	Cornell University	21	483	33

## Data Availability

No new data were created or analyzed in this study. Data sharing is not applicable to this article.
